# Relationship between dynamics of Epstein-Barr virus and immune activation in HIV-1 infected subjects in the HAART era

**DOI:** 10.1186/1758-2652-13-S4-P213

**Published:** 2010-11-08

**Authors:** AM Cattelan, M Zanchetta, L Sasset, R Petrara, R Freguja, K Gianesin, MG Cecchetto, F Cremona, A De Rossi

**Affiliations:** 1 Division of Infectious Diseases, General Hospital of Rovigo, Viale Tre Martiri, Rovigo, Italy; 2 IOV-IRCCS, Padova, Italy; 3 Dept Oncology and Surgical Sciences, Section of Oncology, AIDS Reference Center, University of Padova, Padova, Italy

## Purpose of the study

HAART has greatly modified the course of HIV-1 infection; however, its impact seems to be less favourable on lymphoproliferative disorders associated with Epstein-Barr virus (EBV) than on other AIDS-defining illnesses. The aim of this study was to estimate the relationship between EBV levels and other viro-immunological parameters in HIV-1 infected subjects in the HAART era.

## Patients and methods

164 HIV-1 infected patients (pts) who attended the Infectious Diseases Unit of Rovigo Hospital, from July 2007 to December 2009 were included in this study. 28% of patients had HBV and/or HCV coinfections. HIV-1 RNA in plasma was quantified by COBAS Taqman HIV-1 test. HIV-1 DNA and EBV-DNA in peripheral blood mononuclear cells (PBMC) were determined by real-time PCR. Lipopolysaccharide (LPS), a marker of microbial translocation, was determined in plasma samples using a chromogenic assay (Limolus Amebocyte Lysate). B-cell activation was analyzed by flow cytometry using monoclonal antibodies CD19PerCP, CD86APC, and CD69PE.

## Results

The median (IQR) EBV-DNA load was 41(1-151) copies/105 PBMC. 48% of pts had CD4 >500 cells/µl and 27% had undetectable HIV viral load. The EBV-DNA level was significantly higher in pts with CD4 below 500 cells/µl than in those with CD4 >500cells/µl [72(14-324) vs 18 (1-80) copies/105; p<0.0001] and in pts with detectable HIV-1 RNA than in those with undetectable viremia [49(7-315) vs 17(1-55) copies/105; p=0.001]. Levels of EBV-DNA were higher in the group of pts with CD4 cell counts >500 cells/µl and high HIV-1 viremia (>1000 copies/ml) than in pts with low viremia, regardless of the immunological status [48(5-153) vs 18(1-60); p=0.015). EBV-DNA was also significantly higher in pts with coinfections than in pts with no coinfections [85(10-527) vs 33(1-114); p= 0.003]. Furthermore, pts with high EBV loads (up to 75th percentile) had higher levels of HIV-1 DNA [40(1-132) vs 10(1-76) HIV-DNA copies/105; p= 0.050) and higher levels of LPS [130(88-244) vs 98(81-134) pg/ml; p=0.024) than pts with low EBV loads. B-cell activation in pts with high EBV loads was confirmed by immunophenotyping; two of these pts developed a B-cell lymphoma.

## Conclusions

These findings suggest that HIV-1viremia, other coinfections, and immune activation play an important role in the B-cell stimulation and expansion of EBV-infected cells. Persistent HIV-1 viremia, despite immunereconstitution, may represent a risk factor for the onset of EBV-related cancers.

